# Synchronous Presence of Nasopharyngeal Carcinoma and Marginal Zone (MALT-Type) B-Cell Lymphoma in the Pharynx

**DOI:** 10.4061/2011/340763

**Published:** 2011-05-31

**Authors:** Triantafyllia Koletsa, Georgios Petrakis, Georgia Karayannopoulou, Dimitrios Pappas, Konstantinos Markou, Georgios Karkavelas, Ioannis Kostopoulos

**Affiliations:** ^1^Department of Pathology, Medical School, Aristotle University of Thessaloniki, 54009 Thessaloniki, Greece; ^2^Department of Otorhinolaryngology, Medical School, Aristotle University of Thessaloniki, AHEPA Hospital, 54124 Thessaloniki, Greece

## Abstract

Synchronous malignancy of squamous cell carcinoma and malignant lymphoma in the head and neck region is extremely rare. Nasopharyngeal carcinoma is a nonlymphomatous, squamous cell carcinoma that occurs in the nasopharyngeal epithelium. Reported herein is a unique case of nasopharyngeal carcinoma occurring simultaneously with MALT-type lymphoma in an 83-year-old woman, who complained of deglutition dysfunction. Endoscopic examination of respective organs revealed a submucosal tumour on the posterior wall of pharynx. Biopsy of the hypopharynx was taken and sent for histological examination, which revealed two different neoplasms. Immunohistochemical and molecular analysis confirmed the diagnosis of nasopharyngeal carcinoma coexisting with a MALT-type lymphoma.

## 1. Introduction

Nasopharyngeal carcinoma is a squamous cell carcinoma that occurs in the epithelium of nasopharyngeal mucosa and has been strongly associated with EBV presence. Signs and symptoms are often subtle and nonspecific and thereby may cause a delay in diagnosis, resulting in clinical presentation at an advanced stage of disease. 

MALT-type lymphoma is a low-grade marginal zone B-cell lymphoma affecting various extranodal sites such as mucosal surfaces and parenchymatous organs [1]. MALT-type lymphomas have been noted to arise at different sites of the head and neck region including ocular adnexae, major salivary glands, oral cavity, tonsils, nasopharynx, oropharynx, hypopharynx, larynx, and thyroid gland [2]. Hypopharyngeal involvement is uncommon in comparison to the other sites of the head and neck area [3, 4]. MALT-type lymphoma of the head and neck often presents diagnostic and therapeutic challenges [5, 6].

Taking all the available references into consideration, coexistence of these two different neoplasms has never been reported. Moreover, because of the fact that nasopharyngeal nonkeratinizing carcinoma may have a rich reactive lymphoid substrate, the presence of MALT-type lymphoma may be passed over. Thus, pathologists should be aware of this concurrent neoplasm existence in order to avoid misinterpretation. 

## 2. Clinical History

**Figure 1:**
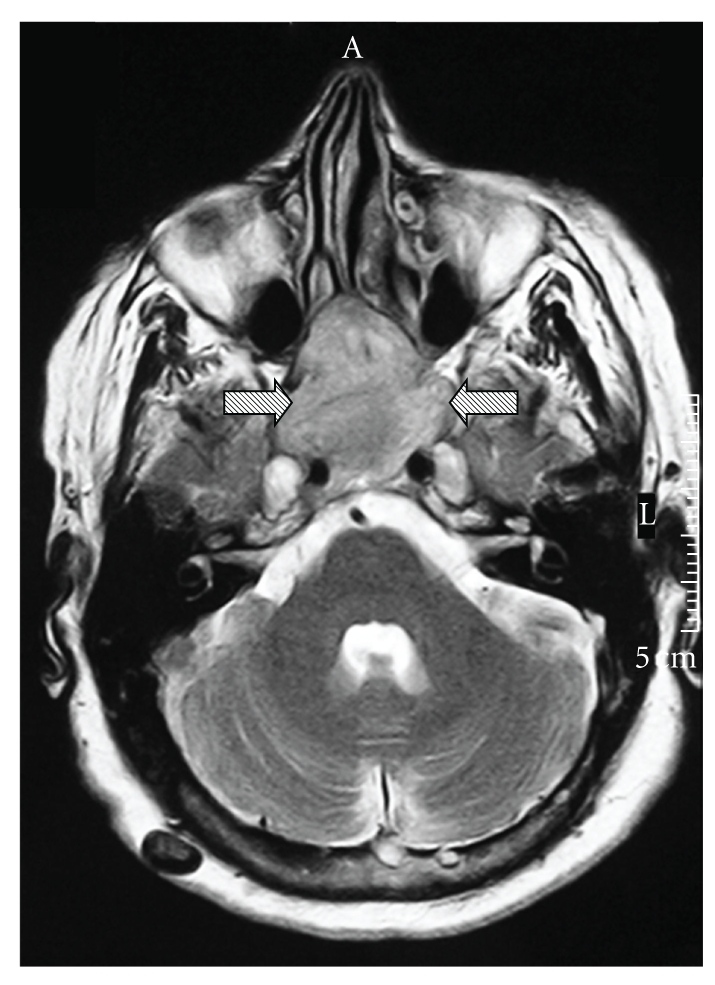
MRI image: a large soft tissue mass (arrows) invades the skull base.

An 83-year-old Caucasian female patient presented with a six-month history of swallowing discomfort and throat pain. On nasopharyngolaryngoscopic examination, a nonulcerative submucosal tumour on the posterior pharyngeal wall was revealed. On MRI images of the head and neck area, a large soft tissue mass of nasopharynx (left and right) was noted (Figure 1), which extended to oropharynx and hypopharynx (right). The mass also invaded skull base, including foramen ovale and foramen rotundum, sphenoid sinus, both major and minor pterygium of sphenoid bone, and pterygopalatine fossa. Therefore, biopsy was taken from the posterior pharyngeal wall and was sent for pathologic examination. The patient deceased before treatment because of extensive disease, which led to respiratory failure, apnea, and heart arrest. 

## 3. Materials and Methods

The specimen was fixed in 10% formalin, and sections were embedded in paraffin blocks. Four micron-thick paraffin sections were stained with hematoxylin and eosin. Unstained paraffin sections were used for immunohistochemical stains. The antibody panel included pankeratin (clone AE1/AE3, DakoCytomation, Glostrup, Denmark), EMA (clone E29, DakoCytomation, Glostrup, Denmark), CAM5.2 (clone 5D3, Novocastra, Newcastle upon Tyne, UK), 34*β*E12 (clone 34*β*E12, DakoCytomation, Glostrup, Denmark), cyclin D1 (clone DSC-6, Novocastra, Newcastle upon Tyne, UK), EGFR (clone 31G7, Zymed, San Francisco, Calif., USA), p53 (clone DO-7, DakoCytomation, Glostrup, Denmark), CD20 (clone L26, DakoCytomation, Glostrup, Denmark), CD45RA (clone 4KB5, DakoCytomation, Glostrup, Denmark), CD45RO (clone UCHL1, DakoCytomation, Glostrup, Denmark), CD3 (clone PS1, Novocastra, Newcastle upon Tyne, UK), CD5 (clone SP19, Spring Bioscience, Inc),CD23 (clone SP23, Spring Bioscience, Inc), CD10 (clone 56C6, Novocastra, Newcastle upon Tyne, UK), and BCL6 (clone PG-B6p, DakoCytomation, Glostrup, Denmark).

For detecting possible infection of neoplastic cells by EBV, we used a chromogenic *in situ* hybridization technique (EBER Histosonda, Cenbimo, Lugo, Spain) according to manufacturer's instructions. Negative and positive controls were used to exclude the possibility of nonspecific staining.

A PCR assay for the detection of a monoclonal rearrangement of the immunoglobulin gene was used. DNA extraction was obtained from paraffin sections, and the PCR technique was performed in accordance to the BIOMED protocols. The products were checked with capillary electrophoresis in genetic analyzer ABI 3130. All reactions were performed with positive and negative controls for each target.

Translocations t(14;18) and t(11;18) were investigated by FISH analysis. The commercially available probes for translocation t(14;18) (LSI IGH/MALT1 t(14;18)(q32;q21) Dual Color, Dual Fusion Translocation Probe, Vysis, USA) and t(11;18) (LSI API2/MALT1 t(11;18)(q21;q22) Dual Color, Dual Fusion Translocation Probe, Vysis, USA) were used. The procedures were performed according to the manufacturer's instructions. 

## 4. Results

**Figure 2:**
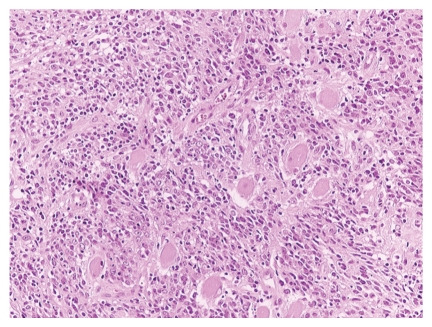
Large round or spindle neoplastic cells arranged in irregular anastomosing islands (HE x200).

**Figure 3:**
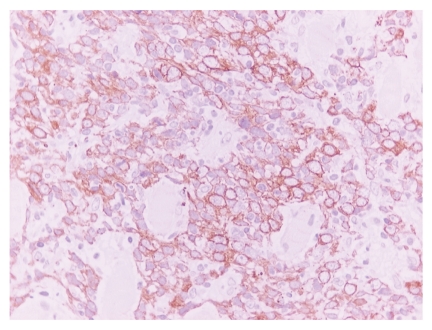
Neoplastic cells show positivity to keratins AE1/AE3 (IHC x400).

**Figure 4:**
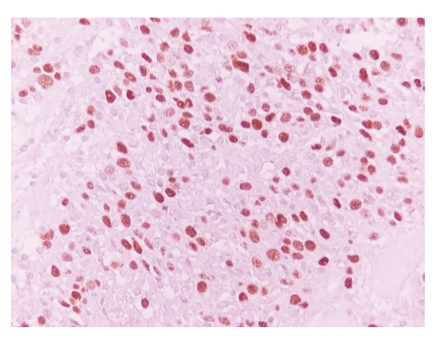
EBER-positive neoplastic cells (x400).

Microscopically, neoplastic cellular nests with a reticular anastomosing pattern were observed. The neoplastic cells were oval or polygonal with round nuclei and quite often with prominent nucleoli (Figure 2). On immunohistochemical examination, these cells were positive for keratins (AE1/AE3, CK8/18, and 34*β*E12) (Figure 3), EMA, Cyclin D1, EGFR, and p53. Moreover, the neoplastic cells were EBER positive by ISH analysis (Figure 4).

**Figure 5:**
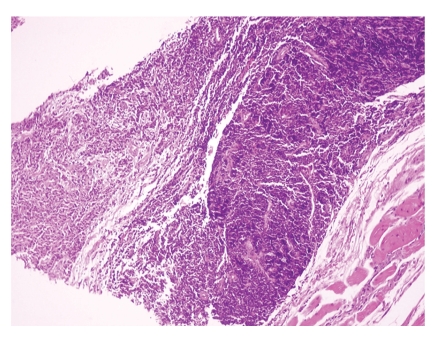
Coexistence of nasopharyngeal carcinoma (left) and MALT-type lymphoma (right) (HE x40).

**Figure 6:**
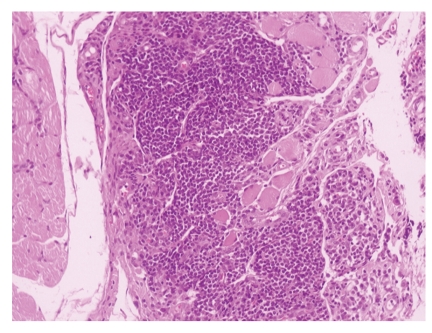
Muscle cells infiltrated by neoplastic lymphoid population (HE x100).

**Figure 7:**
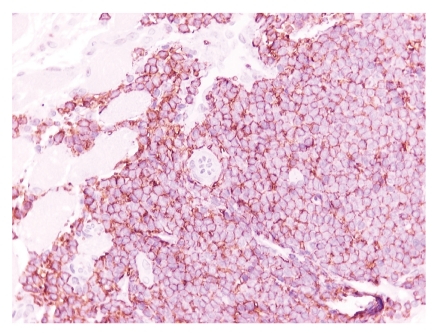
Positivity of neoplastic lymphoid cells to CD20 antibody (IHC x400).

Close beside the above-mentioned carcinoma, a lymphomatous monomorphic population was observed consisting of small-to medium-sized lymphoid cells with pale cytoplasm and slightly irregular nuclei (Figures 5 and 6). The lymphoid cells were localized in the deeper submucosal tissue of hypopharyngeal wall, infiltrating the striated muscle cells (Figure 6). Mitotic figures were usually few. Immunohistochemical stains demonstrated CD20 and CD45RA positivity, a finding consistent with the presence of a B-cell lineage lymphoma (Figure 7). Tumour cells were negative for T-cell markers, such as CD45RO and CD3, although a few reactive T-cells were scattered among the lymphomatous population. Stains for CD5, CD10, BCL6, CD23, and Cyclin D1 were also negative. On PCR analysis a monoclonal IGH gene rearrangement was detected and neither t(14;18) nor t(11;18) translocations were found by FISH analysis.

Based on the above histological, immunohistochemical, and molecular findings, the diagnosis of nasopharyngeal undifferentiated carcinoma coexisting with a marginal zone B-cell lymphoma of MALT-type was set. 

## 5. Discussion

Nasopharyngeal carcinoma is a nonlymphomatous, squamous cell carcinoma occurring in the epithelium of the nasopharynx. According to WHO classification [7], this case belongs to the undifferentiated subtype of nasopharyngeal carcinoma. The fact that 10% of the patients are asymptomatic or present quite often subtle or nonspecific signs and symptoms and the difficulty of making a clinical examination of the nasopharynx lead to a delay in diagnosis, resulting in clinical presentation at an advanced stage of disease [7–9], as happened in our case.

Subclassification of nonkeratinizing nasopharyngeal carcinoma into the undifferentiated and differentiated subtypes has no clinical or prognostic significance [7]. Pathological findings include large cells with round or oval nuclei that show no keratinization. These cells, arranged in solid sheets, irregular islands, discohesive sheets, and trabeculae, are frequently intermingled with lymphoid cells. The density of lymphoid cells varies from scattered to abundant. In case of prominent nonneoplastic lymphoid component, the term lymphoepithelioma and more recently lymphoepithelial carcinoma was used [10]. Neoplastic lymphoid cells in nasopharyngeal carcinoma have never been described.

Lymphomas of the head and neck region are considered as a single group based on their topographical localization in this anatomic area, which is the most common site of extranodal affection by non-Hodgkin's lymphomas. However, MALT-type lymphomas are rare in Waldeyer's ring [11]. Signs and symptoms of a non-Hodgkin's lymphoma may be similar to those of squamous cell carcinoma, and the distinction is made only by biopsy. A respective percentage of patients with head and neck MALT-type lymphoma have a synchronous lymphomatous lesion in another organ [4, 12]. In this case, the importance of facing the nasopharyngeal carcinoma and eventually the patient's decease did not permit the investigation of multiorgan lymphoma involvement. The vast majority of MALT-type lymphomas, and especially those in extragastric sites, are negative for translocations t(11;18) and t(14;18) [13], as noted in this case too.

The coexistence of these two neoplasms raises the question of a common etiologic factor. Nasopharyngeal carcinoma has been associated with EBV infection and quite often, apart from neoplastic epithelial cells, a few scattered bystander lymphocytes are EBV-positive [7, 9, 14]. EBV implication in MALT-type lymphoma development remains doubtful and not well understood [15, 16]. Globally, MALT-type lymphoma is infrequently associated with EBV [15, 17]. In our case, we could not conclude that EBV infection is the reason of the MALT-type lymphoma development, since the neoplastic lymphoid population was EBER negative.

Simultaneous occurrence of carcinoma and MALT-type lymphoma in the same organ has been more frequently reported in stomach [18], since H. pylori is considered to play an aetiological role in both adenocarcinoma and lymphoma. In addition, coexistence of carcinoma and MALT-type lymphoma in other organs has been rarely described in English literature, including scarce cases of thyroid gland [19, 20] and liver [21]. 

## 6. Conclusions

Synchronous presence of squamous cell carcinoma and lymphoma in the head and neck area is extremely rare [22, 23]. Moreover, to the best of our knowledge, this is the first case of nasopharyngeal carcinoma coexisting with MALT-type lymphoma. Since nasopharyngeal carcinoma is quite often accompanied by dense lymphocytic infiltration, we report this case, not only for its rarity but also in order to be acquainted with this coexistence and avoid any misinterpretation.
